# How can formative research inform the design of an iron-folic acid supplementation intervention starting in first trimester of pregnancy in Bangladesh?

**DOI:** 10.1186/s12889-015-1697-2

**Published:** 2015-04-12

**Authors:** Ashraful Alam, Sabrina Rasheed, Nazib UZ Khan, Tamanna Sharmin, Tanvir M Huda, Shams E Arifeen, Michael J Dibley

**Affiliations:** Sydney School of Public Health, The University of Sydney, Sydney, NSW 2006 Australia; International Centre for Diarrhoeal Disease Research, Bangladesh, GPO Box 128, Dhaka, 1000 Bangladesh

**Keywords:** Iron-folic acid, Formative research, Qualitative, Bangladesh

## Abstract

**Background:**

The study objective was to understand community preparedness for iron and folic acid (IFA) supplementation early in pregnancy and to inform the design of a large-scale trial of early introduction of IFA supplementation in rural Bangladesh.

**Methods:**

66 in-depth interviews (pregnant women, husbands, and older women in the household), 20 key-informant interviews, 3 focus-group discussions (community health workers and adolescent female students), and observation of two community-based clinics were conducted.

**Results:**

Most of the women who used IFA tablets during pregnancy reported better health and physical strength after taking them. Women perceived that IFA increased blood volume, leading to foetal nourishment and compensated for blood loss during delivery. However, a culturally informed perceived barrier was the belief that IFA supplementation will increase foetus size, leading to birth complications, hospitalisation, caesarean section and financial burden for the family. Community health workers (CHWs) of BRAC (a non-government organisation) were the main sources of IFA information and supplements, although knowledge of IFA tablets among women’s social networks also helped to make it acceptable. Pregnant women felt that they could start taking IFA during the first trimester of pregnancy if advised by the CHWs. Programme managers and healthcare providers expressed concern about starting IFA supplementation early.

**Conclusion:**

Our study suggests that introduction of IFA supplementation early in pregnancy is feasible with support from CHWs. Promotion of IFA could benefit from efforts to include culturally sensitive reasons for usage; improvement of the CHW training modules; targeted home visits and counselling; and outreach to standardize messages.

## Background

Iron-deficiency is the most widespread nutritional deficiency globally and the most common cause of anaemia during pregnancy [1-4]. It is highly prevalent and most severe in pregnant women, infants, and children, as iron requirement rises during these stages of life [5,6]. Globally, 38% (or about 32 million) of pregnant women were estimated to be anaemic in 2011 [7] and the deaths of 87,321 women worldwide were attributed to iron deficiency [8]. Studies indicate that iron deficiency anaemia in pregnancy is a risk factor for pre-term delivery [5], stillbirth [9], poor neonatal and infant health [10] and maternal death during childbirth [11]. Researchers have also reported that compared to women with normal iron status, anaemic mothers give birth to a higher proportion of low birth weight (LBW) babies [12-14], resulting in higher perinatal morbidity and mortality and higher infant mortality [14]. Anaemia in pregnancy also contributes to intergenerational cycles of poor growth in populations [15], cognitive impairment [6] and losses in human and economic capital [16].

Iron supplementation, nutrition education (to encourage diversified diets), fortification of foods with iron, and de-worming, done alone or in combination, are all approaches widely applied for controlling iron deficiency anaemia during pregnancy [4]. In a review of iron-folic acid (IFA) supplementation during pregnancy, researchers reported a 70% reduction in anaemia at term, a 67% reduction in iron deficiency anaemia, and a 19% reduction in the incidence of low birth weight [1,13]. Researchers have identified lower risks of neonatal mortality in infants for mothers who reported taking antenatal iron-folic acid (IFA) supplements during pregnancy compared to those who did not [17]. In Indonesia a 34% reduction in risk of <5 child death was seen when the mother consumed any IFA supplements during pregnancy [18]. This protective effect was greatest for deaths on the first day of life (60% reduction) followed by deaths during 1–30 days of life (31% reduction), and deaths during the rest of the neonatal period (26% reduction). Folic acid and iron in combination was shown to be more effective than iron or folic acid supplementation alone for the treatment both of anaemia during pregnancy [19] and of neonatal mortality [20].

Recently the World Health Organisation (WHO) recommended a standard daily dose of IFA supplementation throughout pregnancy starting as early as possible to control iron deficiency [21]. A few studies indicated that initiating IFA supplementation earlier in pregnancy may provide increased benefits, such as prevention of iron and folic acid deficiency during pregnancy [22] and reduction of <5 mortality [18]. Despite these recommendations, in many countries such as Bangladesh, women start IFA supplementation after approximately twenty weeks of gestation [22]. The *Shonjibon* trial – a large-scale community-based randomised controlled trial of IFA supplementation in rural Bangladesh – was designed to test the hypothesis that initiation of IFA supplementation in the first trimester will reduce neonatal mortality compared to usual iron-folic acid supplementation programmes. Our study was designed to explore benefits and challenges around IFA supplementation during early pregnancy from both community and service provider perspectives in order to inform the design of the *Shonjibon* trial.

## Methods

### National and local context for IFA supplementation

Anaemia control received a place in Bangladesh’s National Plan of Action for Nutrition from as early as 1997. In 2001 a National Guideline for the prevention and control of iron deficiency anaemia was formulated. The goal of the guideline is to reduce the prevalence of anaemia by one-quarter by 2015 [23]. The strategy to address this goal pays special attention to children, pregnant women, lactating women, adolescent girls, and married women (non-pregnant) with no prescription about timing for starting IFA supplementation. In national maternal health programmes, a standard formulation of IFA tablets with 60 mg of iron is available for distribution through different public health facilities free of charge for daily supplementation as soon as women come to the facilities (usually during their second trimester). Several national non-government organisations (NGOs) and the private sector also provide IFA supplements to pregnant and lactating women in both rural and urban areas of Bangladesh. In the study areas, the facility-based national IFA distribution programme was supplemented by two NGOs - BRAC and *Shabolombi*. The NGO workers made household visits where they counselled pregnant women and sold them various formulations of IFA tablets available in market. Different types of IFA tablets were also available for purchase from local pharmacies.

### Data collection

We collected data using qualitative methods. Data were collected during July–October 2012. The field team consisted of 3 women and 2 men with a master's degree in anthropology or social science, as well as a few years of experience in collecting qualitative data. They were trained and supervised by qualified anthropologists (NK, TS), a medical anthropologist (AA) and a nutritionist (SR).

Four qualitative methods – in-depth interviews, key informant interviews, focus group discussions and observations of the lowest level of health facility (community clinics) were conducted (Table 1).

**Table 1:** **Methods used and sample size**

**Methods**	**Types of respondents**	**Number**
In-depth interviews	Pregnant women	35
	Older women	20
	Fathers	11
Key informant interviews	CHW, program managers, informal care providers, gynaecologists, traditional birth attendants	20
Focus group discussions	Frontline health worker (SS)	2
	Female college students	1
Observation	Community clinic	2

In-depth interviews were used to explore women’s and family members’ perceptions of: cultural norms during pregnancy; birth weight; IFA supplementation; and experience using IFA supplementation during pregnancy. Key informant interviews were conducted to understand the community norms around IFA supplementation and challenges and successes of the existing programme. FGDs were used to understand both perceptions regarding the need for IFA tablets during pregnancy and the state of IFA supplementation programmes. An hour-long observation was conducted in two community clinics to understand how the national IFA programme was being implemented.

### Study locations and populations

We conducted the fieldwork in two villages, one in Mymensing and one in Netrokona– two of the five districts included in the *Shonjibon* Trial– located in north-central Bangladesh. One village in each district was selected for the study. One of the authors (SR) and the formative research field staff consulted the district managers of the BRAC Health Programme to identify one Upazilla (sub-district) from each district in which to conduct the fieldwork. The selected Upazillas best represented their districts in terms of demography, distance from the district office of the NGOs headquarters, and availability of health services from the Government and NGOs. The research team then consulted with the respective NGO Health Programme Managers to select one village in each Upazilla for data collection.

Stratified purposeful and opportunistic sampling [24] strategies were used to select the respondents. The Programme Managers selected two community health supervisors or *Shasthya Kormi*s (SK) from each selected village. *Shasthya Shebikas* or SS (frontline health workers employed by BRAC) who worked under the supervision of the selected SK in the village were interviewed. The pregnant women were selected for the in-depth interviews from the registers of the SKs based on their parity (primipara or multipara) and their use of IFA tablets (user or non-user). All the mothers or mothers-in-law of the pregnant women were also approached for interview. When neither the mother-in-law nor the mother was present, the interviewer interviewed an older female of the family who played a similar role to the mother-in-law or mother.

All the participants of the study provided written consent and the study received ethical clearance from the Ethical Review Committee of the International Centre for Diarrhoeal Disease Research, Bangladesh.

### Data analysis

All the interviews were audio-recorded and transcribed in Bangla for analysis by the researchers themselves. The data obtained were manually coded for emerging themes. Themes were triangulated using data collected through various qualitative methods. Peer debriefing was conducted within the study team to help understand the issues and consolidate the findings.

## Results

### Perceptions related to pregnancy and childbirth

#### Birth weight and size

In the community, people did not aspire for a certain weight for their babies and, therefore, showed no concern about birth weight. No specific terminology for ‘low birth weight’ was found. People recognised a low-birth-weight baby by its appearance and described it as ‘immature’, ‘undeveloped’, ‘small’, or ‘light’. The pregnant women and their mothers-in-law were concerned about having a healthy baby rather than aspiring for a baby of a certain weight. They perceived a ‘normal’, healthy baby to look ‘natural’, be able to feed, cry, and move as expected for a newborn baby. Pregnant women were more concerned about caring for a baby after its birth than by its weight at birth:

*Life and death [of a baby] is in the hands of Allah (sic). Even a small baby born in seven months (sic) [of gestation] can survive if it’s given proper care.* – a pregnant woman

Most husbands desired a large baby with a healthy appearance, while wives wished for smaller babies as it would mean ‘less suffering’ during birth, uncomplicated delivery, and reduce the need for surgery. Discussion among family members about the size and weight of the baby at birth was hindered by perceptions that by articulating aspirations about the unborn child, respondents may bring misfortune to the mother or the infant.

#### Development and nourishment of the foetus

Pregnant women were aware that mother’s dietary intake nourishes the foetus. All pregnant women interviewed perceived that the foetus receives food, blood, and nourishment directly from the mother’s body. One pregnant woman explained:

*Suppose I eat adequate fruits, then the baby in my belly will receive it (sic). If I drink a lot of milk, the baby [in my belly] will get the milk*.

Women were aware of the need to eat normal food during pregnancy. Most pregnant women and elderly women knew of food taboos during pregnancy. Women learned about these ‘harmful’ foods from family members and neighbours. Physical appearance of a food played a role in its description as harmful. For example, coconut and coconut water, if consumed during pregnancy, were said to make the eyes of the baby white (blind). Husbands did not have any knowledge about, or role in, their wife’s diet during pregnancy. They usually deferred to the knowledge of older women in the family. However, some husbands thought that eating too much rice during pregnancy could reduce the size of the baby, as a full stomach left little space for the foetus to grow. This perception was also prevalent among mothers-in-law and mothers of the pregnant women. As one husband explained with a livestock analogy,

*We know if a pregnant woman eats much (sic) the baby inside her tummy will be smaller, for example, if you feed a cow too much food [during pregnancy], it will deliver a small calf*.

#### Medication and movement during pregnancy

Some specific restrictions about taking medicine during pregnancy were mentioned. Pregnant women mentioned that medication for fever and worms during pregnancy could cause abortion and stillbirth. Women said that during pregnancy medicine should be taken according to a doctor’s advice. Here, ‘doctor’ often referred to personnel in the health service delivery system such as SS or SK, without regard to their formal qualifications. Although IFA tablets were considered medicine, pregnant women and their family members relied on the SS, SK and doctors’ advice and hence valued using the IFAs.

*If the doctor knows that you have a baby in your tummy and asks you to take [a medicine] then you take it, otherwise don’t (sic). There is no justification for taking a [bio-]medicine or other kind of medicine, say herbal remedies, on one’s own*. – an older women.

Women believed that going out of the house during certain times of the day and on certain days of the week was harmful for pregnant women. For example, respondents considered noon, sunset, and after the call for evening prayer (*esher aajan*) to be harmful times. Some considered Saturday and Tuesday to be harmful for pregnant women to be out of the house. It was believed that going outside the house during these times or days would pose the danger of being exposed to ‘bad air’ and evil eyes, causing the death of the foetus. Further, it was considered shameful to display the state of pregnancy to men outside the family. These perceptions reduced the mobility of the pregnant women.

#### Benefits of taking IFA supplements

Very few women were aware of the clinical term ‘anaemia’ or how to prevent it. However, many women were aware of the benefits of antenatal intake of iron supplements. Women who used IFA tablets during pregnancy reported more strength, increased blood volume, and reduced physical weakness. The family members of users of IFA tablets perceived that antenatal IFA supplementation increases blood volume and cleans the blood (*rokto baraye* and *rokto porishkar kore*) for pregnant women. There was a common belief that during pregnancy women share their blood with the foetus leaving less blood available for the mother. IFA tablets were believed to make up for this deficiency. As one pregnant respondent said,

*One should take iron tablet [during pregnancy]. When there is a baby in the tummy, the volume of blood is reduced in the body. So iron tablet (sic) should be taken to make up this deficiency.*

Some respondents believed that taking IFA tablets reduced nutrient deficiency and helped recovery from blood loss during pregnancy. According to one elderly woman,

*We are poor people. We can’t afford proper food. We can’t afford fruits as it should be. The amount of blood the baby (foetus) ‘eats’ goes from mother’s body. Mother loses this amount of blood. If she takes iron tablets, she can recover this loss*. (sic)

It is important to note that women who did not take IFA supplements talked about using the supplements if they felt unwell during pregnancy.

Pregnant women's age and parity was a factor for some differences of opinion among about IFA. Younger and primimarous women had more a positive view of IFA supplementation compared to older and multiparous women. Further, a few mothers-in-law and mothers of pregnant women perceived that taking IFA supplements during pregnancy increases the size of the unborn baby which, in turn, can pose an increased risk of birth complications and additional costs because of necessary hospitalisation and surgery. As a result they did not allow their daughters-in-law or daughters to take IFAs.

*…then I visited [my] baper bari* (parental home)*. People there scared me. They said that if I take iron tablet, the baby in my tummy will become bigger and difficult to deliver. –* a pregnant woman

### Experience of taking IFA supplements

Pregnant women who had taken IFA tablets during pregnancy reported positive experiences. They mentioned feeling healthier and stronger after starting the supplements.

*Since I have been using it [IFA tablet] (sic), I don’t feel weakness. I used to feel weak, but now I don’t. –* a pregnant woman

When asked about the kinds of supplements they would like to take, most women preferred capsules to tablets as the tablets did not smell good to them. A few women preferred tablets as they are smaller than capsules and, therefore, easier to swallow. Pregnant women reported experiencing the side effects of IFA supplementation, such as nausea and vomiting, diarrhoea, constipation, and blackened stool. SKs however, mentioned that sometimes women mistook usual pregnancy related nausea and vomiting for side effects of IFA supplementation.

### Sources of IFAs and of information

The SSs and the SKs were the main sources of information about, and supply of, antenatal IFA supplements for pregnant women. During routine home visits, SSs counselled pregnant women about IFA tablets and sold them to those who wanted them. Additionally, almost all of the pregnant women mentioned hearing about IFA tablet consumption during pregnancy from other women in their social networks and media (radio and television), which helped them to accept the IFA tablets. Adolescent female students talked of learning about the use of IFA supplements during pregnancy from their social networks, demonstrating that knowledge about IFA tablets during pregnancy was widespread among women. As one respondent said,

*I heard about iron tablet from my sister when she was taking it, and she heard about it from her mother-in-law (sic).*

Pregnant women were advised about taking IFA tablets from informal (allopathic, homeopathic, and traditional medical practitioners, and drug sellers) and formal healthcare providers (gynaecologists). However, they did not mention receiving counselling about the need for IFA from these sources. Interestingly, none of the women interviewed talked about the primary government facility or Community Clinics as a source of information or supply of IFA tablets. During our observation of Community Clinics, two pregnant women attended the facilities with health problems. None of them received counselling about IFA tablets. One woman was given three tablets to take and asked to come back for more. When we asked why only three tablets were given, the health worker told us that she wanted to make sure that the pregnant woman did not waste the medication. She also told us that she likes to provide the medication to all women who visited the clinic, but was worried that there was not enough stock to provide a month’s supply to each woman.

Although SSs advised the pregnant women to take IFA supplements, they lacked a nuanced understanding of why IFA tablets are needed during pregnancy. They themselves were unsure about the link between IFA tablets and delivery complications. As a result they often simply followed the instructions from their supervisors (SKs) and sold the supplements to the pregnant women without trying to convince them through counselling. Further, SSs did not have adequate training in terms of managing the side effects of IFA tablets. If some women complained about side effects, the SSs asked the women to reduce the frequency of the supplementation and if that did not work they advised the women to stop taking the supplements. This management strategy was based on what the SS felt made most sense, rather than being based on medical training.

### Influence on decision-making

According to the pregnant women, BRAC SSs and SKs were very influential in their decisions about taking IFA tablets. Most women knew the SSs and SKs and trusted them as providers of reliable health information. Women mentioned that they would take IFA if the *Shasthya kormi* (health workers, referring to the SS and SK) advised them to do so. Women’s IFA supplementation coincided with household visits by the health workers.

*If ShasthyaKormi Apa tells that [a pregnant woman] should take iron tablets, they will take it. … But they visit from the fifth month [of pregnancy] (sic). If they visited earlier, we would start [iron tablets] earlier. – a pregnant woman.*

At the household-level, mothers-in-law had very strong influence in deciding whether pregnant women would consume IFA supplements. These older women were both sources of advice and support for the pregnant women, as well as being in many cases the gatekeepers. For many families mothers-in-law were in charge of family finances; when it came to buying IFA tablets from the SSs, this role came into play. Husbands had little role in decisions regarding pregnancy care; pregnancy was considered a female domain and the husbands usually deferred to the opinions of older women in the family and that of the pregnant women themselves. When it came to money matters, however, husbands were involved and were considered the most important decision-makers.

### Barriers to early antenatal IFA supplementation

Our data did not indicate strong resistance from the community to taking IFA supplements early in pregnancy, if supported by SS and SK. A few women however, expressed concern about the link between intake of IFA supplements during pregnancy and large babies. This concern was expressed by both pregnant women and mothers-in-law. However, these concerns were linked to consumption of IFA tablets generally, rather than to the timing of IFA consumption specifically. As one pregnant woman said,

*If you eat much [during pregnancy] the baby in the tummy gets bigger. If you take iron tablet your baby will also get bigger and create complications for delivery.*

Although the common practice is to start the supplementation around the fifth month of the pregnancy, this practice reflected the usual time of the first antenatal visit in the community and programme decisions made by the BRAC Health Programme. Most pregnant women and their family members, as well as health workers interviewed in this study, indicated that earlier initiation of antenatal IFA supplementation would not be opposed by the community. However, specific concerns about early supplementation were mentioned by the health care providers and programme managers. The programme managers were concerned that the community would hold the programme responsible for any premature births or miscarriages among women who took IFAs early in pregnancy on health workers’ advice. As indicated by a participant SK during FGD:

*We forbid [the women] to take iron tablet during the first three months, [because] if any abortion happens during this period they will think that it happened due to intake of iron tablet (sic).*

A local gynaecologist when interviewed was reluctant to prescribe IFAs during the first trimester of pregnancy as she worried about losing a patient due to the side effects of IFA tablets. During the first stage of pregnancy, the doctor preferred to provide only folic acid supplements.

*I don’t prescribe iron-folic acid in the first trimester. As I said, vomiting tendency sustains for three months. After that vomiting tendency reduces, pregnancy hormones reduce. Then she (pregnant woman) can accept it (IFA supplements). If she vomits, iron deficiency will increase.* – a gynaecologist

## Discussion and conclusions

This paper explores the cultural and programmatic feasibility of providing IFA tablets early in pregnancy in rural Bangladesh. Understanding potential feasibility will benefit programmes both in Bangladesh and in other low- and middle-income countries [3]. Table 2 contains a list of programmatic implementations of the qualitative insights for intervention on early initiation of IFA supplements during pregnancy.

**Table 2:** **Programmatic implications for intervention**

**Areas of interest**	**Constraints and barriers**	**Favourable factors**	**Opportunities for intervention**
Perception of birth weight	• No desired minimum or maximum birth weight and size for their unborn baby.	Women desire a ‘normal’ and ‘healthy’ baby.	Training SS and SKs to:
	• Discussions about the condition of an unborn child are perceived to be harmful.		• convey messages about the importance of normal birth weight and role of IFA and
			• discuss the risks associated with low birth weight and how anaemia is linked to low birth weight.
Perception of size of the baby	Concerns that a large baby would create delivery complications.		Promote awareness that a big baby does not necessarily create birth complications if the mother remains healthy during pregnancy and appropriate antenatal care is received.
Perception of antenatal iron-folic acid intake	• Perceived to increase the size of the foetus resulting in increased birth complications and potential surgery.	• IFA is perceived to increase volume and quality of blood, which helps women recover from blood loss during childbirth, and retain their physical strength.	Training SS and SKs to enable them to:
	• Higher parity women are less positive about iron-folic acid intake.	• Younger and first-time pregnant women tend to follow advice of health workers sincerely.	• take available opportunities such as community events to improve awareness of antenatal IFA intake in community.
	Negative impression among few older females.		• counsel pregnant women about impact of IFA on the growth of foetus in greater detail.
			• use existing favourable cultural reasoning in the promotion of antenatal IFA supplementation.
			• target older and multiparous pregnant women for counselling
			• include older women in the household in the counselling sessions.
Experience IFA intake	• Side effects are experienced by pregnant women.	• Many women do not consider these side effects very serious.	• Use a formulation of iron-folic acid with minimal smell
	• Smell of IFA supplements is difficult to stand for many pregnant women.	• No concern about colour of the supplement.	• Train SS and SKs to provide better counselling about possible side effects and management of side effects
Sources of information on iron-folic acid	• In rare cases, pregnant women receive negative and discouraging information about IFA from their social network.	• Many currently pregnant women heard about IFA at younger age and before they were pregnant from the pregnant women in the family and neighbourhood.	• SS and SK of BRAC should be used to distribute and counsel about IFAs.
		• SS and SK are the main sources of information on antenatal IFA intake.	• The SS and SKs should be trained to deal with negative information from outside the trial zone.
Influence of family members on decision-making	Husbands have little concern about their wife taking IFA and consider it a female domain.	Mothers-in-law have the strongest influence in decision-making.	Mothers-in-law should be counselled in the few initial home visits after IFA supplements are supplied.
Concerns of IFA early in pregnancy	• SS/SK: Concerns that the community might hold the overall BRAC health programme responsible for any miscarriage/abortion that happens to a women who was taking iron-folic acid supplements early in pregnancy on their advice.	• Widespread opinion exists among pregnant women in favour of starting IFA intake as early as a BRAC SS/SK advises.	• Distribute iron-folic acid supplement and provide instructions to use through BRAC SSs/SKs who have already established their trustworthiness in the community.
		• BRAC Managers at Upazila level are confident about the ability of SS/SKs to motivate their clients to start IFA according to the Programme’s decision.	• Address the community concerns about IFA supplementation in the CHW training.
Medical pluralism	Multiple sources of IFA supplements and information exists	Pregnant women usually visit private practitioners only when they are sick.	Maintain cordial relationship and organise periodical stakeholders meetings with providers outside BRAC Health Program to align messages about early IFA supplementation
Costs	Women wanted to have access to free IFA supplements.	The *Shonjibon* project has resources for free provision of IFA supplements.	Provide IFA supplements free of cost to reduce economic burden on the family.

Our study revealed that there is no local term for ‘low birth weight’ in Bangla (the language spoken by the study community). Although this could indicate a lack of expectation around a specific or minimal birth weight, families did express concern about having a healthy infant. This community-level lack of concern regarding birth weight is in direct contrast to scientific literature, where low birth weight is associated with increased morbidity and mortality among infants [14]. On the other hand, large babies were associated with birth complications and increased financial burden to the family. Similar concerns about large babies were reported by other researchers [25-27]. In IFA supplementation programmes, therefore, it is important to inform the community about the health risks associated with low-birth-weight babies and the role of IFA tablets in reducing incidence of low birth weight. The pregnant women also need to be reassured about the perceived link between IFA supplementation and large babies as concern about delivering large infants was shown in both Bangladesh and other countries [25,27,28].

From interviews with women and health workers it is apparent that front-line health workers need better training for awareness and management of side effects of IFA supplements. Prior studies conducted in Bangladesh [29] and Cambodia [30] have concluded that efforts to further reduce side effects may not be successful in improving compliance of antenatal iron supplementation. However, those studies did not take into account the importance of increasing awareness about possible side effects and management of the side effects. Our study and other studies suggests that providing women with more information about possible side effects and educating them about managing those side effects can potentially improve compliance [25].

Our study supported the findings of other studies in low and middle resource setting about a lack of knowledge on maternal anaemia and beliefs restricting certain nutritious foods that could benefit healthy pregnancies [28,30,31]. These findings demonstrate the necessity of providing better and comprehensive counselling for pregnant women, and making the IFA tablets accessible. Using CHWs who live in the community and are trusted by community members could enhance programme effectiveness, as has been seen in other maternal and neonatal health programmes [32,33] and IFA supplementation programmes [34,35]. It is important to note that acceptability of the health worker to the community does not guarantee the delivery of adequate information to the patients [25]. Experience from South East Asia and Africa shows [30,36] that health workers require proper training to effectively counsel pregnant women.

Positive perceptions and cultural beliefs about IFA intake – such as increased volume and quality of blood and physical well-being – can promote adherence to the supplementation regimen. Interestingly, these same perceptions have been found in India, Senegal, and several other low and middle resource settings [26,28,37]. Although the SS and SK are the prime sources of information about iron supplements and their role in pregnant women’s health for most respondents in our study area, some younger women had received information from older women – particularly their elder sisters-in-laws, prior to marriage or pregnancy. In addition to being sources of information, family members play a role in deciding whether or not a pregnant woman will take the supplements, as seen in others settings also [26,37]. In our study, we found strong influence of the mothers-in-law and weak influence of husbands in women’s decision-making about IFA intake. Based on the evidence about the family members’ influence on women’s knowledge and decision-making, our study recommends the inclusion of family members, particularly older women and mothers-in-law, during the counselling of the pregnant women.

Our study showed older and multiparous pregnant women were less likely to take IFA tablets and listen to advice. This finding is contradictory to a few other studies [30,38], but is supported by recent Bangladesh Health and Demographic Survey results [39]. It is important, therefore, that any programme aiming to increase compliance with the taking of IFA tablets target pregnant women based on age and parity.

In the study, community antenatal care and IFA tablets were received from multiple sources, similar to other settings [31,35], which indicates a need to standardize health information about IFA tablets in order to increase the coverage and acceptance of IFA supplementation. This is particularly relevant if IFA supplementation is started in the first trimester of pregnancy. In our study, neither formal doctors nor NGO healthcare providers reach out to pregnant women before the second trimester. And both groups expressed concerns about prescribing IFA supplementation in early pregnancy. It is important, then, to train both NGO workers and local private practitioners about the value of early IFA supplementation, so that all providers trusted by the community are supportive of IFA programmes. To acquire this support efforts should be made to communicate regularly and maintain good relationships with the providers outside the programme [31,40]. For the implementer of early IFA supplementation programmes, it is crucial to train their managers and field workers about the need for early IFA supplementation, and adequately address their concerns regarding all aspects of the programme. A strong effort to create buy-in from the personnel actually implementing the programmes will be crucial for the success of the programme.

Our study has some limitations that would be considered when interpreting the results of the study. As a formative and qualitative study, the results are not intended to be representative of all rural settings in Bangladesh. Further validation would be needed to assess programmatic feasibility of providing IFA supplementation early in pregnancy in the rural communities with specifically distinct characteristics such as non-Bengali speaking ethnic groups.

## Abbreviations

BRAC: Bangladesh Rural Advancement Committee
CHW: Community Health Worker
ICDDR,B: International Centre for Diarrhoeal Disease Research, Bangladesh
FGD: Focus Group Discussion
IFA: Iron-Folic Acid
LBW: Low birth weight
SK: *Shasthya Kormi*
SS: *Shasthya Sebika*
NGO: Non-governmental Organisation
WHO: World Health Organisation
